# Metabolic engineering of *Pseudomonas putida* for increased polyhydroxyalkanoate production from lignin

**DOI:** 10.1111/1751-7915.13481

**Published:** 2019-08-30

**Authors:** Davinia Salvachúa, Thomas Rydzak, Raquel Auwae, Annette De Capite, Brenna A. Black, Jason T. Bouvier, Nicholas S. Cleveland, Joshua R. Elmore, Jay D. Huenemann, Rui Katahira, William E. Michener, Darren J. Peterson, Holly Rohrer, Derek R. Vardon, Gregg T. Beckham, Adam M. Guss

**Affiliations:** ^1^ National Bioenergy Center National Renewable Energy Laboratory Golden CO 80401 USA; ^2^ Biosciences Division Oak Ridge National Laboratory Oak Ridge TN 37831 USA; ^3^ Present address: University of Calgary Calgary AB Canada; ^4^ Present address: National Renewable Energy Laboratory Golden CO 80401 USA; ^5^ Present address: Pacific Northwest National Laboratory Richland WA 99354 USA

## Abstract

Microbial conversion offers a promising strategy for overcoming the intrinsic heterogeneity of the plant biopolymer, lignin. Soil microbes that natively harbour aromatic‐catabolic pathways are natural choices for chassis strains, and *Pseudomonas putida* KT2440 has emerged as a viable whole‐cell biocatalyst for funnelling lignin‐derived compounds to value‐added products, including its native carbon storage product, medium‐chain‐length polyhydroxyalkanoates (*mcl*‐PHA). In this work, a series of metabolic engineering targets to improve *mcl*‐PHA production are combined in the *P. putida* chromosome and evaluated in strains growing in a model aromatic compound, *p*‐coumaric acid, and in lignin streams. Specifically, the PHA depolymerase gene *phaZ* was knocked out, and the genes involved in β‐oxidation (*fadBA1* and *fadBA2*) were deleted. Additionally, to increase carbon flux into *mcl*‐PHA biosynthesis, *phaG, alkK, phaC1* and *phaC2* were overexpressed. The best performing strain – which contains all the genetic modifications detailed above – demonstrated a 53% and 200% increase in *mcl*‐PHA titre (g l^−1^) and a 20% and 100% increase in yield (g *mcl*‐PHA per g cell dry weight) from *p*‐coumaric acid and lignin, respectively, compared with the wild type strain. Overall, these results present a promising strain to be employed in further process development for enhancing *mcl*‐PHA production from aromatic compounds and lignin.

## Graphical abstract

In this work, a series of metabolic engineering targets to improve mcl‐PHA production were combined in the *P. putida* chromosome and evaluated in strains growing in a model aromatic compound, p‐coumaric acid, and in lignin streams. Specifically, the PHA depolymerase gene (*phaZ*) and genes involved in β‐oxidation (*fadBA1* and *fadBA2*) were deleted, while *phaG*, *alkK*, *phaC1*, and *phaC2* were overexpressed to increase carbon flux from fatty acid biosynthesis to mcl‐PHA production. This strain demonstrated an increase in mcl‐PHA titer (g l^−1^) and yield (g mcl‐PHA per g cell dry weight) from p‐coumaric acid and from lignin compared to the wild‐type strain, resulting in a promising strain to be employed in further process development for enhancing mcl‐PHA production from aromatic compounds and lignin.

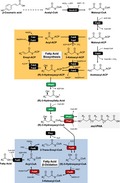

## Introduction

Lignocellulosic biomass offers a source of renewable carbon that can reduce reliance on fossil fuels, reduce greenhouse gas emissions and build a foundation for a sustainable bioeconomy. Recent studies have demonstrated that the co‐production of hydrocarbon fuels from sugars and chemicals from lignin streams increases the value proposition for biorefinery processes (Davis *et al*., 2013; Ragauskas *et al*., 2014; Corona *et al*., 2018). By leveraging natural host metabolic capabilities and applying genetic engineering techniques, carbon from complex and heterogeneous substrates, such as lignin, can be funnelled into single products (Linger *et al*., 2014; Beckham *et al*., 2016). Of particular relevance to this work, the production of oleochemicals by native and engineered microbes has gained increased attention in the last decade due to the demand for more sustainable fuels and consumer and industrial products (Pfleger *et al*., 2015). An example of these oleochemicals is medium‐chain‐length polyhydroxyalkanoates (*mcl*‐PHAs). These polymers can be used in the production of biodegradable plastics, medical devices and chemical and material precursors (Philip *et al*., 2007; Linger *et al*., 2014; Mozejko‐Ciesielska and Kiewisz, 2016; Prieto *et al*., 2016; Chen and Jiang, 2018).

**Figure 1:**
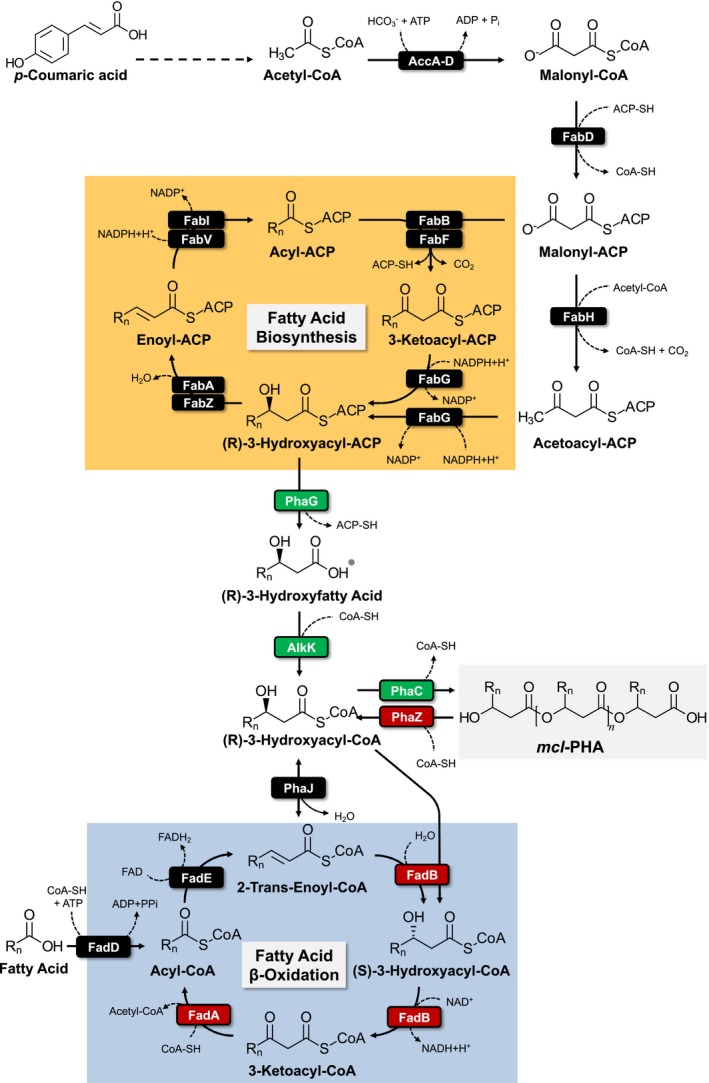
The *mcl*‐PHA production pathway in *P. putida* KT2440 via fatty acid biosynthesis and competing fatty acid β‐oxidation pathway. Red boxes indicate genes targeted for deletion, and green boxes indicate genes targeted for overexpression. AccA‐D, acetyl‐CoA carboxylase; FabD, malonyl CoA‐ACP transacylase; FabH, 3‐ketoacyl‐ACP synthase; FabG, 3‐ketoacyl‐ACP reductase; FabA and FabZ, 3‐hydroxyacyl‐ACP dehydratase; FabI and FabV, enoyl‐ACP reductase; FabB and FabF, 3‐oxoacyl‐ACP synthase; PhaG, hydroxyacyl‐ACP acyl‐transferase; AlkK, acyl‐CoA‐synthase; PhaC1 and PhaC2, PHA polymerases; PhaZ, PHA depolymerase; PhaJ, R‐specific enoyl‐CoA hydratase; FadB, enoyl‐CoA hydratase**/**3‐hydroxyacyl‐CoA dehydrogenase; FadA, 3‐ketoacyl‐CoA thiolase; FadE, acyl‐CoA dehydrogenase; FadD, long‐chain acyl‐CoA synthetase.

The individual monomers comprising *mcl*‐PHAs can range from C6 to C14 in chain length. *mcl*‐PHAs are biosynthetic polyesters that can be produced from a wide range of carbon sources in many bacteria. Some of the most well studied *mcl*‐PHAs producers are fluorescent Pseudomonads (Madison and Huisman, 1999; Prieto *et al*., 2007). Among these Pseudomonads, *Pseudomonas putida* KT2440 naturally produces *mcl*‐PHAs as a carbon storage compound in scenarios of carbon excess and nutrient limitation (de Eugenio *et al*., 2010). This bacterium is genetically tractable and metabolically diverse, with many advantageous features for biorefinery processes (Nikel and de Lorenzo, 2014). *P. putida* can produce *mcl*‐PHAs from multiple carbon sources such as fatty acids, which directly undergo β‐oxidation, or from sugars and aromatic compounds, which are subjected to fatty acid *de novo* biosynthesis (Prieto *et al*., 2016) (Fig. 1). Specifically, metabolic intermediates in the fatty acid biosynthetic pathway are converted to (R)‐3‐hydroxyacyl‐ACP, which is hydrolyzed to a free hydroxy‐fatty acid and subjected to CoA ligation, via PhaG and AlkK, respectively, to produce (R)‐3‐hydroxyacyl‐CoA. Similarly, the β‐oxidation intermediate 2‐trans‐enoyl‐CoA can be hydrated to generate (R)‐3‐hydroxyacyl‐CoA by PhaJ (Tsuge *et al*., 2000). These CoA monomers can then be polymerized into *mcl*‐PHAs via the PHA synthases, PhaC1 and PhaC2 (Ren *et al*., 2009). The reverse process, PHA degradation, can also occur in scenarios of complete carbon depletion or sudden nutrient increases and is conducted via the PHA depolymerase, PhaZ (de Eugenio *et al*., 2010).

Metabolic engineering has been applied to improve *mcl*‐PHA production through various routes (Chen and Jiang, 2018) such as (i) shutting down competing pathways (β‐oxidation), (ii) overexpressing the PHA synthesis operon (via plasmid or chromosomal integration) with different ribosome binding sites (RBS) and/or promoters, (iii) enhancing NADH or NADPH supply for PHA synthesis, (iv) engineering cell morphology to increase cell size and (v) eliminating the ability to consume PHAs. For instance, to decrease the flux of PHA pathway intermediates to the fatty acid β‐oxidation pathway, the genes *fadA* and *fadB* were deleted in *P. putida* which resulted in a 2.5‐fold increase in *mcl*‐PHA production (wt.% basis) when grown on nitrogen‐rich medium supplemented with heptanoate and octanoate (Wang *et al*., 2011). In route (ii) above, the overexpression of *phaG* in the PHA synthesis operon of *Pseudomonas jessenii* resulted in a fourfold increase in *mcl*‐PHA accumulation (wt.% basis) from phenylacetic acid (Tobin *et al*., 2007). The effect of overexpressing other genes in combination with *phaG* on *mcl‐*PHA production was also tested in *E. coli*. The expression of *phaG* and *phaC1*(STQK) resulted in minimal *mcl*‐PHA production from glycerol (0.9 mg l^**−**1^), while the expression of *phaG*,* phaC1* (STQK) and *alkK* increased *mcl*‐PHA accumulation to 25 mg l^**−**1^ when grown in the same conditions (Wang *et al*., 2012). Another strategy to enhance PHA accumulation is to eliminate the degradation of the polymer through the deletion of the PHA depolymerase gene, *phaZ*. This deletion was evaluated in *P. putida* and resulted in a 1.9‐fold increase in *mcl‐*PHA titre (g l^**−**1^) and 1.3‐fold increase on PHA production (wt.% basis) when grown on octanoate under nitrogen‐limited conditions (Cai *et al*., 2009) and in a 47% PHA increase (wt.% basis) when grown on glycerol as a sole carbon source (Poblete‐Castro *et al*., 2014). De Eugenio *et al*. (de Eugenio *et al*., 2010) also reported a similar result in a comparable background strains after 48 h of incubation utilizing octanoate as carbon source while Cai *et al*. (Cai *et al*., 2009) showed a significant improvement in the knockout strain after 5 days of cultivation.

Most of the work reported to date on metabolic engineering in Pseudomonads and other organisms to improve *mcl*‐PHA production utilizes carbohydrates and oil sources as substrates, while only a few studies describe the use of aromatic compounds or lignin as a carbon source (Table 1). Furthermore, many reported strains employ plasmid‐based approaches, which limits applicability during scale‐up due to the need for antibiotic use to retain plasmids. In this study, we sought to engineer a base strain of *P. putida* to produce *mcl‐*PHAs from aromatic compounds and lignin using genomic integration of DNA throughout. We demonstrate improved *mcl*‐PHA production from *p*‐coumaric acid (*p*‐CA) and a lignin stream that contains this aromatic compound as a major carbon source (Rodriguez *et al*., 2017). The combination of overexpressing *mcl‐*PHA synthesis genes and deleting *mcl*‐PHA degradation genes increased *p*‐CA and lignin conversion into *mcl*‐PHA. Overall, this work presents a robust base strain whose background can be utilized for further process development and/or engineering to produce new compounds that utilize similar biosynthetic pathways.

**Table 1:** Literature describing *mcl*‐PHA production from lignin‐derived aromatic compounds and lignin streams by native and engineered bacteria

Strain	Substrate	Antibiotic	Cultivation mode	Cultivation time (h)	CDW (mg ml^−1^)	*mcl*‐PHA (mg l^−1^)	*% mcl*‐PHA yield (g per g CDW)	References
Native strains								
*P. putida* JCM13063	Vanillic acid	–	Batch, flask	72	210	Traces^c^	< 1	Tomizawa *et al*. (2014)
*P. putida* GPO1	p‐Coumaric acid	–	Batch, flask	72	270	Traces^c^	< 1	Tomizawa *et al*. (2014)
*P. putida* KT2440	p‐Coumaric acid		Batch, flask	72	378	160	41	This study
*P. putida* KT2440	p‐Coumaric acid	–	Batch, flask	48	470	160	34	Linger *et al*. (2014)
*P. putida* KT2440	Ferulic acid	–	Batch, flask	48	436	170	39	Linger *et al*. (2014)
*P. putida* KT2440	Lignin‐containing stream (corn stover) d		Batch, flask	78	399	35	8.8	This study
*P. putida* KT2440	Lignin‐containing stream (corn stover) d	–	Bioreactor, FB	48	787	252	32	Linger *et al*. (2014)
Engineered strains								
*P. putida* A514 _DVJ4C1_	Kraft lignin	T, G	Batch, flask	40	b	70	b	Lin *et al*. (2016)
*P. putida* A514 _ΔphaJ4/phaC1_	Vanillic acid	T, G	Batch, flask	b	b	b	73.5	Lin *et al*. (2016)
*P. putida*_Δxyl_alkKphaGC1_	Vanillic acid	T	Batch, flask	50	715	246	34	Wang *et al*. (2018)
*P. putida* KT2440^a^	Lignin‐containing stream (corn stover) d	a	a	a	5300	1000	17.6	Liu *et al*. (2017)
*P. putida* AG2162	p‐Coumaric acid	–	FB, flask	72	483	241	50	This study
*P. putida* AG2162	p‐Coumaric acid	–	FB, flask, HCD	85	1758	953	54.2	This study
*P. putida* AG2162	Lignin‐containing stream (corn stover)^d^	–	Flask, batch	78	654	116	17.7	This study

CDW, cell dry weight; FB, fed‐batch; G, gentamicin; HCD, high‐cell density; T, tetracycline.

**a**. The strain is not specified. In the materials and methods section, the authors specify the use of a native strain (in batch mode) while in their results authors stress the use of an engineered strain (in fed‐batch mode).

**b**. Not reported.

**c**. Not clear if authors analyzed *mcl*‐PHAs or only polyhydroxybutyrate [P(3HB)].

**d** .The origin and preparation of these lignin streams is different in each case.

## Results and discussion

### Genetic modifications of *P. putida* to improve mcl‐PHA accumulation

To improve *mcl*‐PHA accumulation, *P. putida* KT2440 was genetically engineered to (i) eliminate *mcl*‐PHA depolymerization, (ii) decrease flux of *mcl*‐PHA pathway intermediates to fatty acid degradation and (iii) increase carbon flux from fatty acid chain elongation to *mcl*‐PHA production (Fig. 1). To eliminate *mcl*‐PHA depolymerization, the gene encoding the PHA depolymerase (*phaZ*; PP_5004) was deleted, resulting in strain AG2102 (Table 2). To decrease 3‐hydroxyacyl‐CoA flux towards fatty acid β‐oxidation, two previously identified chromosomal copies of the enoyl‐CoA hydratase/3‐hydroxyacyl‐CoA dehydrogenase (*fadB*) and 3‐ketoacyl‐CoA thiolase (*fadA*) genes (Liu *et al*., 2007) were deleted. The *fadBA1* genes are encoded at loci PP_2136‐2137 in a putative two‐gene operon. The second *fadBA* genes are clustered within loci PP_2214‐2217, where FadB is encoded at two separate coding regions – PP_2214 (3‐hydroxyacyl‐CoA dehydrogenase) and PP_2217 (enoyl‐CoA hydratase) – while FadA is encoded at PP_2215. This putative operon also encodes an acyl‐CoA dehydrogenase (*fadE*; PP_2216). These two gene clusters, PP_2136‐2137 and PP_2214‐2217, were deleted in strain AG2102, resulting in strain AG2228. Finally, to increase carbon flux from 3‐hydroxyacyl‐ACP to *mcl*‐PHAs, an additional, codon‐optimized copy of the hydroxyacyl‐ACP thiolase (*phaG*; PP_1408), the hydroxyacyl‐CoA synthase (*alkK*; PP_0763), and the two PHA polymerases (*phaC1* and *phaC2*; PP_5003 and PP_5005), were integrated into the chromosome of AG2228 and overexpressed using the constitutive *P*_*tac*_ promoter. These genes were inserted into the chromosome by replacing an acetaldehyde dehydrogenase (*aldB*; PP_0545) that is presumably not involved in aromatic catabolism, resulting in strain AG2162.

**Table 2:** *P. putida* KT2440 genotypes and strain designations. Plasmids and strains used in this work were constructed using standard protocols as described in the Appendix S1 and as reported before (De Boer *et al*., 1983; Johnson and Beckham, 2015; Kvitko and Collmer 2011, Marx, 2008).

Strain	Genotype
AG2102	*P. putida* KT2440 Δ*phaZ*
AG2228	*P. putida* KT2440 Δ*phaZ ΔfadBA1 ΔfadBAE2*
AG2162	*P. putida* KT2440 Δ*phaZ ΔfadBA1 ΔfadBAE2 ΔaldB::P*_*tac*_ *‐phaG‐alkK‐phaC1‐phaC2*

### Strain evaluation for mcl‐PHA production from the model aromatic compound p‐CA

**Figure 2:**
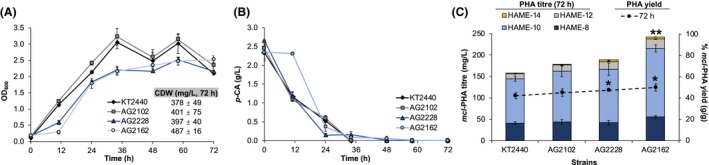
Production of *mcl*‐PHAs from *p*‐CA. A. Optical density at 600 nm (OD_600_) as a function of time and cell dry weight (CDW) at the end time point. B. *p*‐CA consumption profiles. C. *mcl*‐PHA titres and composition (bars), detected via depolymerization and derivatization to hydroxyacyl methyl esters (HAMEs), and *mcl*‐PHA yields (g *mcl*‐PHA per g CDW) at 72 h (black circles) in four different strains. Results present the average of biological triplicates and error bars show the standard deviation. A statistical analysis (t‐test) was also performed for *mcl‐* PHA titres and yields between the wild type and the engineered strains. *Significant difference at 95% confidence (see yields). **Significant difference at 99% confidence (see titres). Batch shake flask cultivations were performed in nitrogen‐limited modified M9 minimal medium (pH 7.2) containing 0.13 g l^**−**1^ (= 1 mM) (NH_4_)_2_ SO_4_ and 2 g l^**−**1^ *p*‐CA in triplicate. Cells were then washed in M9 (without carbon and nitrogen source) and the flasks were inoculated to an initial OD_600_ of ~ 0.1 and incubated for 72 h. Samples for *mcl*‐PHA analysis were washed twice with distilled water and lyophilized for cell dry weight (CDW) measurements and PHA extraction. For *mcl*‐PHA production and composition analysis, samples were derivatized in BF_3_‐methanol and quantified by gas chromatography‐mass spectroscopy (GC‐MS) as described in Appendix S1.

To determine if *mcl*‐PHA accumulation was affected by the genetic modifications, these strains were grown in nitrogen‐limited medium containing 2 g l^**−**1^ *p‐*CA (12.2 mM) and 0.13 g l^**−**1^ (NH_4_)_2_SO_4_ (1 mM). AG2228 and AG2162 presented longer growth lags than KT2440 and AG2102 (Fig. 2A). Despite these initial growth profiles, *p*‐CA maximum utilization rates were higher in the former strains (i.e. 0.15 ± 0.00 g l^**−**1^ h^**−**1^ in AG2162 and 0.10 ± 0.03 g l^**−**1^ h^**−**1^ in KT2440) and *p*‐CA was nearly depleted at a similar time (48 h) in all the strains (Fig. 2B). *mcl*‐PHA titres (mg l^**−**1^) at the sample collection time (72 h) only increased significantly in the engineered strain AG2162 (242.0 ± 9.8 mg l^**−**1^) when compared to the wild type (157.8 ± 10.2 mg l^**−**1^) (Fig. 2C). In all cases, 3‐hydroxydecanoate (C10) and 3‐hydroxyoctanoate (C8) were the major *mcl*‐PHA components produced, while 3‐hydroxydodecanoate (C12) and 3‐hydroxytetradecanoate (C14) were present in minor abundance, as expected (Fig. 2C). The proportions of the four constituents were similar in the tested strains as well (as a percentage of total *mcl*‐PHAs produced, 22‐26% C8, 66% C10, 7‐10% C12 and 1‐2% C14). The PHA yields (g *mcl*‐PHA per g CDW) in AG2228 and AG2162 also exhibit significant increases compared with the wild type. Particularly, the yield increased from 41.9 ± 2.8% in wild type to 47.3 ± 1.2 and 49.8 ± 3.5% in AG2228 and AG2162, respectively.

Although the deletion of *phaZ* did not improve titres or yields, this genetic background will be still advantageous in further process development, which requires longer cultivations subjected to carbon‐starvation. While the further deletion of *fadBA1* and *fadBAE2* in AG2228 led to a statistically significant increase in yields when compared to the wild type, titres did not improve. A similar result was previously reported in a different *P. putida* strain when only deleting *fadA* and *fadB*, but utilizing fatty acids as a carbon source (Wang *et al*., 2011). The overexpression of *phaG* alone was previously reported not to affect *P. putida mcl*‐PHA production from phenylacetic acid (Tobin *et al*., 2007). Separately, overexpression of *phaC1* combined with *phaJ4* was sufficient to increase *mcl*‐PHA accumulation from vanillic acid in a plasmid‐bearing *P. putida* strain (Lin *et al*., 2016) (Table 1) while in *E. coli,* overexpressing the genes encoding PhaC and AlkK was necessary to enhance *mcl*‐PHA accumulation from glycerol (Wang *et al*., 2012). Even though the present study has not evaluated single overexpressed genes, we demonstrate that the selected gene combination (gene knockouts and gene overexpression integrated into the genome) significantly improves carbon flux from *p*‐CA into *mcl*‐PHA biosynthesis in *P. putida*.

### Evaluation of mcl‐PHA production by AG2162 under different culture conditions

**Figure 3:**
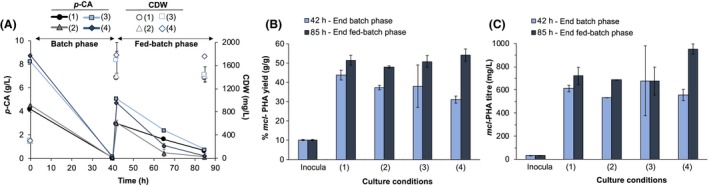
Production of *mcl‐* PHA by AG2162 in fed‐batch mode at different C (*p*‐CA, g l^**−**1^):N ((NH_4_)_2_ SO_4_, mM) ratios and concentrations in the batch phase, (1) 4:0, (2) 4:1, (3) 8:2, (4) 8:4, and fed‐batch phase (1) 2.5: 0, (2) 2.5:0, (3) 5:0, (4) 5:0. (A) Consumption of *p*‐CA and CDW, (B) *mcl*‐PHA yields, and (C) *mcl*‐PHA titres. The ‘inocula’ case corresponds to the seed culture data before inoculation. Results show the average of two biological replicates. Error bars present the absolute difference from the biological duplicate. These experiments were conducted in shake flasks. AG2162 was precultured from glycerol stocks in modified M9 medium containing 2 g l^**−**1^ *p*‐CA and non‐limiting nitrogen (10 mM (NH_4_)_2_ SO_4_) for 24 h. The preculture was then washed twice in M9 medium (without carbon or nitrogen), and inoculated at an OD_600_ of 4 in modified M9 medium containing different carbon (*p*‐CA):nitrogen ((NH_4_)_2_ SO_4_) ratios in the combinations mentioned above. When *p*‐CA was depleted (42 h), a pulse of 2.5 or 5 g l^**−**1^ *p*‐CA was also applied to the combination (1,2) or (3,4) respectively. Flasks were incubated at 30°C and 300 rpm for 85 h and samples were taken at 42 and 85 h to evaluate CDW and PHA production.

Carbon (C)‐to‐nitrogen (N) ratio (de Eugenio *et al*., 2010) and cell density (Davis *et al*., 2015) are known to affect *mcl‐*PHA accumulation in *P. putida*. Thus, to obtain higher *mcl*‐PHA titres and yields than those obtained in the previous experiment (Fig. 2C), we evaluated AG2162 for *mcl*‐PHA production at different C:N ratios and concentrations in a high‐cell density, fed‐batch, shake flask experiment. The experimental setup consisted of a batch phase containing either 4 or 8 g l^**−**1^ *p*‐CA as a carbon source with (NH_4_)_2_SO_4_ at different concentrations yet still nitrogen‐limited and a fed‐batch phase where the feeding contained only *p*‐CA as a carbon source without any supplementary nitrogen (see Fig. 3 legend).

*p*‐CA was depleted at the end of the batch phase and its concentration was < 0.75 g l^**−**1^ at the end of the fed‐batch phase in all the cultivation conditions (Fig. 3A). Maximum *p*‐CA utilization rates (calculated after the feeding pulse) decreased at higher C:N ratios. Specifically, when nitrogen was absent from the media during the batch phase (case 1), *p*‐CA utilization rate was 0.06 ± 0.00 g l^**−**1^ h^**−**1^ and, at the lowest C:N (case 4), the rate was 0.16 ± 0.01 g l^**−**1^ h^**−**1^. Utilization rates were the same (0.11 ± 0.01 g l^**−**1^ h^**−**1^) in cases 3 and 4, which correspond to the same C:N ratio but at different initial substrate concentrations. Regarding CDW (Fig. 3A), the highest values were observed in case 4, at the lowest C:N ratio. The *mcl*‐PHA yields were similar at the end of the fed‐batch phase in all cases (between 48% and 54% with errors up to 3.2%) (Fig. 3B), and case 4 presented the greatest CDW. Therefore, *mcl‐*PHA titres were also higher in the latter case, up to 953 ± 44 mg l^**−**1^ (Fig. 3B). The average yields at the end of the batch phase increased at the highest C:N ratio (case 1, without nitrogen added). However, since nitrogen starvation limits cell growth, the titres were ultimately similar to those found under other culture conditions (cases 3 and 4). These results suggest that the C:N ratios evaluated in this study do not have a critical effect on *mcl*‐PHA yields if produced in fed‐batch mode and high‐cell density cultivations. However, that ratio is critical to enhance cell biomass and thus titres and productivity.

### Production of mcl‐PHAs from a soluble and process‐relevant lignin‐rich stream

**Figure 4:**
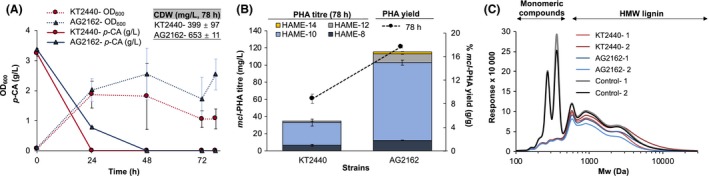
Performance of wild type and AG2162 *P. putida* strains in a process‐relevant soluble lignin stream. A. Bacterial density and *p*‐CA utilization over time, (B) *mcl*‐PHA titres (mg l^**−**1^) and composition (bars), detected via depolymerization and derivatization to hydroxyacyl methyl esters (HAMEs), and *mcl*‐PHA yields (g *mcl*‐PHA per g CDW) at 78 h (black circles) and (C) GPC lignin profiles after the bacterial treatments and in non‐inoculated lignin controls. Results show the average of two biological replicates with error bars representing the absolute difference. The cultivation conditions were the same as those presented in the legend of Fig. 2 except that in this case, the cultivations were performed in 250 ml baffled flasks containing 50 ml of medium (modified M9 plus 75% sterile soluble lignin stream). Non‐inoculated lignin cultures were used as a control. For lignin content, a compositional analysis was performed in freeze‐dried lignin supernatants according to the procedure in NREL LAP/TP‐510‐42618 (Sluiter *et al*., 2006). For molecular weight, gel permeation chromatography (GPC) analysis was also conducted on freeze‐dried samples (30 mg) as described before (Salvachúa *et al*., 2016). The analysis of *p*‐CA in non‐lignin containing media was analyzed by high performance liquid chromatography (HPLC) on an Agilent 1100 series equipped with a Phenomenex Rezex RFQ‐Fast Fruit H^+^ column and cation H^+^ guard cartridge at 85°C, using 0.01 N sulphuric acid as a mobile phase at a flow rate of 1.0 ml min^−1^ and a diode array detector scanning at 325 nm. The analysis of aromatic compounds in lignin cultures was conducted as previously described (Salvachúa *et al*., 2018).

As demonstrated above, wild type and engineered *P. putida* strains are able to convert the lignin‐derived product *p*‐CA to *mcl*‐PHAs effectively. Thus, to finalize this study, we also tested the ability of both strains to convert a heterogeneous lignin stream that contains *p*‐CA, ferulic acid and high molecular weight lignin as major carbon sources, to *mcl*‐PHAs. This lignin comes from the solid fraction generated after enzymatic hydrolysis of pretreated corn stover (Chen *et al*., 2016). Then, it is further washed with water (to remove sugars) and solubilized via base‐catalyzed depolymerization (Rodriguez *et al*., 2017; Salvachúa *et al*., 2018). Lignin solubilization was approximately 53% (lignin content in soluble stream/lignin content in initial solid stream) and contained ~ 4 g l^**−**1^ of *p*‐CA and 0.1‐0.2 g l^**−**1^ ferulic acid (as major aromatic compounds) from an initial total lignin content of approximately 22 g l^**−**1^. Wild type *P. putida* and strain AG2162 were grown in the lignin liquor (75% v/v containing 1 mM (NH_4_)_2_SO_4_) and reached stationary phase between 24 and 48 h, likely due to the total consumption of readily accessible carbon sources and/or nitrogen (Fig. 4A). Strain AG2162 increased the *mcl*‐PHA yield by ~ 100% compared with the wild type (17.7 ± 0.2 vs. 8.9 ± 0.8% respectively) and titre by 3.3‐fold (116 ± 35 vs. 35 ± 5 mg l^**−**1^ respectively) (Fig. 4B) which demonstrates the robustness of AG2162 and the increased carbon flux into *mcl*‐PHA biosynthesis even in complex lignin streams. The main hydroxyacid species accumulated in both strains was again 3‐hydroxydecanoate. However, unlike the proportions observed in Fig. 2C, 3‐hydroxyoctanoate was lower in these lignin cultures, representing 10% and 18% of the hydroxyacids in AG2162 and KT2440, respectively (Fig. 4B), instead of 22‐26%. We also analyzed the lignin molecular weight profile by gel permeation chromatography (GPC) at the end of the cultivations (78 h). Low molecular weight lignin (indicated as monomeric aromatic compounds in Fig. 4C) disappeared after the bacterial treatments, which aligns with the total *p*‐CA depletion shown in Fig. 4A. As observed in previous work (Salvachúa *et al*., 2015), both strains also decreased the high molecular weight lignin content, although that decrease is more evident in AG2162 cultivations (Fig. 4C) which suggests the conversion of oligomeric lignin. To confirm if high molecular weight lignin is metabolized by strain AG2162 to a higher extent than KT2440, we also analyzed the lignin content at the end of the bacterial treatments. Lignin utilization was 23.5 ± 1.7% and 18.3 ± 1.5% in AG2162 and KT2440, respectively, which verifies the GPC observations. Overall, these results corroborate that AG2162 is a robust and improved *mcl*‐PHA production strain compared with KT2440 from both pure aromatic compounds, such as *p*‐CA, and a process‐relevant lignin stream.

Culture conditions (Davis *et al*., 2015), carbon sources (Cai *et al*., 2009) and volume ratios (v_media_:v_flask_) (Poblete‐Castro *et al*., 2014) are critical parameters in *mcl*‐PHA production. Considering the number of variables, quantitative comparison of *mcl*‐PHA production studies on an equivalent basis is challenging. In fact, comparisons become more complicated when using a heterogeneous substrate as lignin since, in many cases, lignin streams contain carbon sources other than aromatic compounds (e.g. acetic acid and sugars) that can lead to the production of *mcl*‐PHAs (Linger *et al*., 2014), or contain very different lignin concentrations [e.g. 10 g l^**−**1^ (Salvachúa *et al*., 2015) to 30 g l^**−**1^ of lignin (Rodriguez *et al*., 2017)]. In addition, considering the increased titres obtained in fed‐batch mode from *p*‐CA (Fig. 3) as well as the *mcl*‐PHA titres (1 g l^**−**1^) achieved from a lignin stream in fed‐batch mode in a recent publication (Liu *et al*., 2017) (Table 1), it is likely that *mcl*‐PHA titres from the current lignin stream could be further improved by using a different feeding strategy. However, in this study we did not pursue optimizing titres from lignin because the main limitation currently faced in valorizing lignin is its low content of bioavailable monomeric aromatic species and carbon to the production hosts (Beckham *et al*., 2016). Nevertheless, it is worth highlighting that the lignin stream utilized in this study contains up to 15% of monomeric species (mainly *p*‐CA) (Rodriguez *et al*., 2017; Salvachúa *et al*., 2018), which is already a reasonable concentration to be upgraded.

Overall, while there is extensive space to improve the conversion of aromatic compounds and lignin to *mcl*‐PHAs through process development in bioreactors, our results suggest that strains developed here can be a reasonable starting platform to efficiently convert lignin‐derived aromatic compounds into different value‐added molecules that are derived from fatty acid biosynthesis (e.g. fatty alcohols, ketones, chemically‐functionalized *mcl*‐PHAs). Furthermore, the AG2162 background can also be utilized for further pathway engineering to increase mcl‐PHA titre, rate and yield by increasing flux into fatty acid biosynthesis in future work.

## Conflict of interest

None declared.

## Supporting information


**Appendix S1**. Materials and methods.
