# Metabolic engineering of *Pseudomonas putida* for increased polyhydroxyalkanoate production from lignin

**DOI:** 10.1111/1751-7915.13547

**Published:** 2020-04-01

**Authors:** Davinia Salvachúa, Thomas Rydzak, Raquel Auwae, Annette De Capite, Brenna A. Black, Jason T. Bouvier, Nicholas S. Cleveland, Joshua R. Elmore, Anna Furches, Jay D. Huenemann, Rui Katahira, William E. Michener, Darren J. Peterson, Holly Rohrer, Derek R. Vardon, Gregg T. Beckham, Adam M. Guss

**Affiliations:** ^1^ National Bioenergy Center National Renewable Energy Laboratory Golden CO 80401 USA; ^2^ Biosciences Division Oak Ridge National Laboratory Oak Ridge TN 37831 USA; ^3^ Present address: University of Calgary Calgary AB Canada; ^4^ Present address: National Renewable Energy Laboratory Golden CO 80401 USA; ^5^ Present address: Pacific Northwest National Laboratory Richland WA 99354 USA

In the originally published article by Salvachúa et al. (2019), the author Anna Furches was accidentally omitted from the byline of manuscript, ‘Metabolic engineering of *Pseudomonas putida* for increased polyhydroxyalkanoate production from lignin’ Volume 13, Issue 1, Pages 290–298. The byline should read as given above.

The authors sincerely apologize for the confusion caused by this error.

