# Supplementary material for: Metabolic engineering of Pseudomonas putida for increased polyhydroxyalkanoate production from lignin
Source: Microb Biotechnol. 2019 Aug 30;13(1):290–8. doi: 10.1111/1751-7915.13481 (PMC6922519; doi:10.1111/1751-7915.13481)
Supplement: Supplementary file 1 — Appendix S1. Materials and methods. [file MBT2-13-290-s001.docx]

**Supplementary File 1. Materials and Methods**

**Plasmid and strain construction:**

The construction of plasmids and strains in this work followed standard protocols. Enzymes and reagents used for cloning were purchased from New England Biolabs (Ipswich, MA) unless otherwise specified. Primers synthesized by Integrated DNA Technologies (IDT, Coralville, IA) and Phusion High-Fidelity DNA Polymerase 2X Master Mix were used in all PCR reactions. Plasmid isolation and purification was performed using QIAprep spin miniprep kits (QIAGEN, Germantown, MD). Genes targeted for overexpression were codon optimized using Gene Designer (DNA 2.0, Newark, CA) and synthesized as gBlocks by IDT. Plasmids were constructed using Gibson Assembly Master Mix according to the manufacturer's guidelines. Briefly, approximately 1,000 bp regions of homology upstream and downstream of the gene of interest were PCR amplified and cloned into pK18mobsacB (Kvitko and Collmer, 2011) using Gibson Assembly, generating deletion plasmids pPhaZ_del, pFadBA1_del, and pFadBAE2_del. For chromosomal integration and *P_tac_*-driven (de Boer et al., 1983) overexpression of an additional copy of codon-optimized *phaG*, *alkK*, *phaC1*, and *phaC2*, gBlocks were PCR amplified and cloned between approximately 1000 bp regions of homology flanking *aldB* (PP_0545), resulting in plasmid pPhaZ_del::PhaG_AlkK_PhaC. Gibson reactions were electroporated into *E. coli* 10G (Lucigen, Middleton, WI) for validation and plasmid propagation. Transformants were grown on LB medium supplemented with 50 μg/mL kanamycin as required for plasmid maintenance. Correct assembly of fragments was verified using restriction digests and sequencing. Plasmid maps and sequence files are provided in Supplementary File 2. *P. putida* KT2440 (ATCC 47054) was engineered using the kanamycin and *sacB* system for selection and counter-selection (Marx, 2008). To make the cells electrocompetent, cells were grown to an optical density at 600 nm (OD_600_) of 0.5-0.7 at 30°C, 225 rpm shaking speed, centrifuged at 4°C, washed twice in ice-cold water and once in ice-cold 10% glycerol, and resuspended in 10% glycerol to a 1/100 of the culture's original volume (Johnson and Beckham, 2015). Cells were transformed immediately or stored at -80°C for future use. Cells (50 μL) were mixed with 500 ng of plasmid DNA in a pre-chilled electroporation cuvette, and electroporated at 1.6 kV, 25 uF, 200Ω. Cells were allowed to recover in 250 μL of SOC recovery medium for 1-2 h at 30°C. Cells were then grown overnight on LB agar plates containing 50 μg/mL kanamycin and re-streaked the following day to ensure culture purity and integration of the plasmid. Colonies were then re-streaked on YT+25% sucrose plates (10 g/L yeast extract, 20 g/L tryptone, 250 g/L sucrose, and 18 g/L agar) for sucrose counter selection to select for loss of the *sacB* gene. Colonies were then re-streaked on YT+25% sucrose plates, and subsequently picked into LB medium. Isolates were also screened on LB agar plates containing 50 μg/mL kanamycin to ensure that they no longer were resistant to kanamycin. Deletion/integration events were verified using primers below.

| **GIBSON ASSEMBLY PRIMERS** | |
| --- | --- |
| **pPhaZ_del** |  |
| Gib_PhaZ_up_fwd | ctctagagtcgacctgcaggcatgcaAGCAGACCTTCATCATCAGC |
| Gib_PhaZ_up_rev | ccgcagctgtt**actagt**GCACGTGACTCTTGGGTG |
| Gib_PhaZ_down_fwd | aagagtcacgtgc**actagt**AACAGCTGCGGCCTGACA |
| Gib_PhaZ_down_rev | acgttgtaaaacgacggccagtgccaCCGGCGCAATTGCTTCTT |
|  |  |
| **pFadBA_del** |  |
| Gib_FadBA_up_fwd | ctctagagtcgacctgcaggcatgcaATGTCCTTCATGCGCGGC |
| Gib_FadBA_up_rev | cgctacgcga**actagt**CAACTGATCTCCACGATATGGAAG |
| Gib_FadBA_down_fwd | tggagatcagttg**actagt**TCGCGTAGCGGGACAGCAG |
| Gib_FadBA_down_rev | acgttgtaaaacgacggccagtgccaGGCCCACAGCAGTGGCGA |
|  |  |
| **pFadBAEx_del** |  |
| Gib_FadBAEx_up_fwd | ccagtcacgacgttgtaaaacgacggccagtgccaagcttAGCTGGGTATCACCAACCTG |
| Gib_FadBAEx_up_rev | TGGGATCCattctaGTCGACaggcatCTCGAGTCTAGAGAGTACTTTCCTTTCAGACGCT |
| Gib_FadBAEx_down_fwd | ACTCTCTAGACTCGAGatgcctGTCGACtagaatGGATCCCAGTGGAAGCAAATTCGCA |
| Gib_FadBAEx_down_rev | tagctcactcaggaaacagctatgacatgattacgaattcCACATCGACTCGGCTATTCA |

| **DELETION/INTEGRATION VERIFICATION PRIMERS** | | **Expected size (bp)** | |
| --- | --- | --- | --- |
| **Δ*phaZ*** |  | **WT** | **Del** |
| PhaZ_Ext_F | AGTACAGCCCCATCACCGAG | 3118 | 2266 |
| PhaZ_Ext_R | ACTTCACCACCGTCCAGCA |  |  |
| PhaZ_Int_F | TCTTTAACGGCATCGGC | 308 | 0 |
| PhaZ_Int_R | TGGCACCATTACCGCA |  |  |
|  |  |  |  |
| **Δ*fadBA*** |  |  |  |
| FadBA_Ext_F | GATGCGCCGTGTTCAGAG | 5479 | 2130 |
| FadBA_Ext_R | ACGCTCAACCACCAGCTTC |  |  |
| FadBA_Int_F | GTCTTCGATGCCACCGTG | 712 | 0 |
| FadBA_Int_R | GTTACCGGTCATGATCGCC |  |  |
|  |  |  |  |
| **Δ*fadBAEx*** |  |  |  |
| FadBAEx_Ext_F | TTGGGCTTACGGCTTGTATT | 7774 | 1759 |
| FadBAEx_Ext_R | ACACCCGACCCTATCATCAC |  |  |
| FadBAEx_Int_F | GACCTCAAGAGCCTGACTGC | 966 | 0 |
| FadBAEx_Int_R | GCCGTGGATATTGACCTTGT |  |  |

*mcl*-PHA production and composition analysis

For *mcl*-PHA production, cells were grown in modified M9 minimal medium (pH 7.2) containing 6.78 g/L Na_2_HPO_4_ x 7 H_2_O, 3 g/L KH_2_PO_4_, 0.5 g/L NaCl, 2 mM MgSO_4_, 0.1 mM CaCl_2_, 5 mg/L FeSO_4_ x 7H_2_O, 0.13 g/L (= 1 mM) (NH_4_)_2_SO_4_, and 2 g/L *p*-CA. Cultivations containing *p*-CA were performed in triplicate in 2 L baffled flasks containing 600 mL of medium. The seed culture was inoculated from glycerol stocks and grown overnight at 30°C, 225 rpm, in LB medium. Cells were then washed in M9 (without carbon and nitrogen source) and the flasks were inoculated to an initial OD_600_ of ~0.1. Cultures were incubated at 30ºC and 225 rpm for 72-78 h. Samples for *mcl*-PHA analysis were taken at the end time point (72 or 78 h), centrifuged at 8,000 rpm for 15 min, and the cell pellets were washed twice with distilled water and lyophilized for cell dry weight (CDW) measurements and PHA extraction. For *mcl*-PHA composition analysis, 10-30 mg of lyophilized cell biomass was added to a GC vial. To track derivatization, 0.2 mg of benzoic acid was spiked to each sample as an internal surrogate. Samples were derivatized by adding 1 mL of BF_3_/MeOH to the GC vial, which was sealed, shaken, and placed in a heating block at 80°C overnight. Vials were then removed from the heating block and allowed to cool to room temperature. Vial contents were pipetted into a 10 mL volumetric flask and the vial residual was rinsed twice with dichloromethane (DCM) before filling the flask to 10 mL total with additional DCM. The 10 mL solution was transferred to a polytetrafluoroethylene (PTFE) capped vial and ~3 mL of water was added to form a bi-phase and wash out residual BF_3_ to the aqueous layer. The DCM layer (~2 mL) was then transferred into another GC vial containing Na_2_SO_4_ and Na_2_CO_3_ to dry and neutralize any remaining BF_3_. The dried and neutralized solutions were syringe filtered (0.2-μm PTFE) into fresh GC vials for analysis. The resulting hydroxyacid methyl esters (HAMEs) were quantified by gas chromatography mass spectroscopy (GC-MS) using an Agilent 7890A GC equipped with a 5975C MSD (Agilent Technologies, Santa Clara, CA). The GC was outfitted with an Agilent DB-Wax column (30 m × 0.25-mm id, 0.25-μm film), and helium (1 mL/min column flow) was used as the carrier gas. The injector volume was set to 1 μL using an Agilent auto-sampler. The GC/MS method consisted of a front inlet temperature of 250ºC, MS transfer line temperature of 250°C, and scan range from 35 m/z to 550 m/z. The oven was held at 35ºC for 5 min, then ramped at 15°C/min until 225°C, held at 225°C for 2 min, then ramped again at 15^º^C/min until 250°C, and then held at 250°C for 2 minutes. C8, C10, C12, and C14 hydroxyacids were obtained from Sigma Aldrich, derivatized, and used to determine the GC-MS instrument response to construct standard curves and quantify *mcl-*PHAs from samples. O-terphenyl (AccuStandard) was employed as an internal standard to correct for GC-MS response changes within a run.
